# Two Cases of Late-Onset Anti-NMDAr Auto-Immune Encephalitis After Herpes Simplex Virus 1 Encephalitis

**DOI:** 10.3389/fneur.2020.00038

**Published:** 2020-02-18

**Authors:** Guillaume Dorcet, Marie Benaiteau, Chloé Bost, Catherine Mengelle, Fabrice Bonneville, Guillaume Martin-Blondel, Jérémie Pariente

**Affiliations:** ^1^Département de Neurologie, Hôpital Pierre Paul Riquet, CHU de Toulouse, Toulouse, France; ^2^Laboratoire d'Immunologie, Institut Fédératif de Biologie, CHU de Toulouse, Toulouse, France; ^3^INSERM U1043 – CNRS UMR 5282, Centre de Physiopathologie Toulouse-Purpan, Toulouse, France; ^4^Laboratoire de Virologie, Institut Fédératif de Biologie, CHU de Toulouse, Toulouse, France; ^5^Service de Neuroradiologie, Hôpital Pierre Paul Riquet, CHU de Toulouse, Toulouse, France; ^6^INSERM: ToNIC, Toulouse NeuroImaging Center, Université de Toulouse, Inserm, Université Paul Sabatier, Toulouse, France; ^7^Service de Maladies Infectieuses et Transmissibles, Bâtiment Urgences Réanimation Médecine, CHU de Toulouse, Toulouse, France

**Keywords:** herpetic meningoencephalitis, herpes simplex, autoimmune encephalitis, anti-NMDA antibodies, cerebrospinal fluid

## Abstract

**Context:** Encephalitis due to herpes simplex virus 1 (HSV-1) was described as a potential trigger for the development of anti-N-methyl-D-aspartate receptor (NMDAr) auto-immune encephalitis (AIE) within a few days to a few weeks after the infection.

**Methods:** We assessed clinical, radiological, and biological diagnoses process, treatment response, and evolution.

**Cases Reported:** We report here cases of a 71-year-old man and a 57-year-old woman presenting anti-NMDAr AIE, respectively, 12 and 7 months after HSV-1 encephalitis. In both cases, the onset was brisk, and the symptoms were mainly neuropsychiatric (paranoid delirium, Capgras, and Cotard syndromes) and cognitive, with anterograde amnesia. Relapse of HSV encephalitis, epilepsy, and paraneoplastic neurologic syndromes were excluded. The clinical response to first-line treatments composed of intravenous immunoglobulins and high-dose corticosteroids was poor, whereas significant improvement was noticed after rituximab induction.

**Conclusion:** Post-herpetic anti-NMDAr AIE could arise several months after infection. Clinicians must be aware of this possibility, particularly if cognitive and/or psychiatric symptoms occurred after a remitting period. In our two cases, only rituximab was associated with clinical improvement.

## Background

Anti-NMDAr related encephalitis is a well-described dysimmune entity of the central nervous system and the most frequent of the auto-immune encephalitis (AIE) (1). Frequently idiopathic, two other etiologic spectrums are identified: first, as paraneoplastic manifestation, and second, as post-infectious neurological complication (2). *Herpesviridae*, and particularly herpes simplex virus-1 (HSV-1), are the most frequent identified viruses associated with anti-NMDAr encephalitis (2–5). In the reported cases and series, AIE arises soon after cerebral infection, between 5 and 75 days post-infection (2–5). We report here two cases of patients presenting with an anti-NMDAr encephalitis diagnosed, respectively, 7 and 12 months after an HSV-1 encephalitis.

## Methods

MRI imaging was performed on three Tesla machines (Magnetom Skyra, Siemens Healthcare, Erlangen, Germany and Achieva 3.0T, Philips, Amsterdam, The Netherlands), and electroencephalograms (EEG) were performed and read on Deltamed software (Natus, Middleton, Wisconsin, USA). Real-time PCR (RT-PCR) was done using in-house method with primers directed to HSV-2 DNA polymerase gene. The HSV genotype was analyzed by melting curves. The difference in melting temperature was due to differences in the sequences of HSV-1 and -2: 57–58°C for HSV-1 and 67–69°C for HSV-2 (6). The presence of autoantibodies was assessed by indirect immunofluorescence on rat hippocampus slices (EuroImmun, Lübeck, Germany) and confirmed by cell-based assay on HEK293 cells expressing GluN1a subunit of the NMDAR (EuroImmun, Lübeck, Germany). Written informed consent was provided by the participants for the publication of this case report. Patient 2 provided written informed consent after recovering for any identifiable information or images contained within the manuscript to be published. The same consent concerning patient 1 was provided by his wife.

## Results

### Case 1

**Figure 1:**
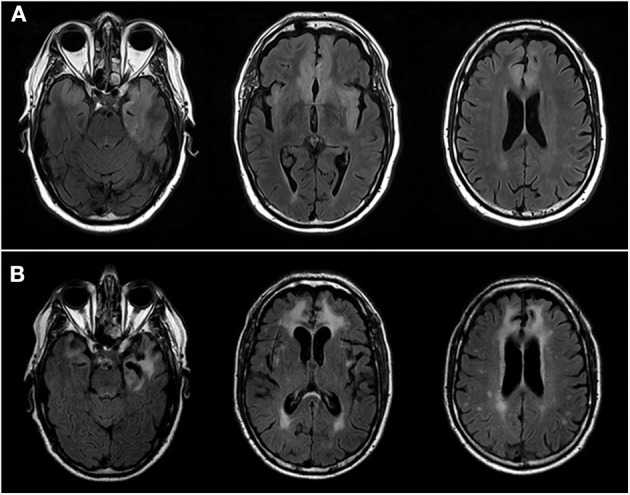
3T Brain MRIs. **(A)** Patient N°1 MRI at the acute phase of the herpetic meningoencephalitis. Axial images showing hypersignal of the anterior, intern, left, temporal lobe including uncus and hippocampus, left insula and bilateral, ventromedial, orbito-frontal and cingulate anterior lobes. **(B)** Patient N°1 MRI at the time of AIE. Axial FLAIR images showing sequels of herpetic meningoencephalitis, including extension of previously describe lesions and necrosis of the medial, anterior, left, temporal lobe. Of note, increase of vasculoderenerative subcortical, bilateral lesions.

A 71-year-old man was hospitalized in our medical center in May 2017 because of febrile, akinetic mutism and several partial, motor, epileptic seizures. He had a medical history of treated hypertension, cured prostate adenocarcinoma, and gout. A brain MRI revealed a T2 hypersignal of the left, medial, anterior, temporal lobe and the bilateral, orbito-frontal lobes (Figure 1A). Cerebro-spinal fluid (CSF) showed lymphocytic pleocytosis with 195 leukocytes/mm^3^ including 96% lymphocytes, increased total proteins 0.72 g/l (normal < 0.5 g/l), decreased glucose 0.4 mmol/l, (normal 3.33–4.44 mmol/l) and real-time PCR was positive for HSV-1. The patient was treated by intravenous acyclovir for 3 weeks and long-term, oral levetiracetam (500 mg twice a day). His medical state improved slowly but he was able to return home in June 2017, after 4 weeks in a rehabilitation center. He kept a partial apathy but was autonomous for common daily tasks and was partially nosognosic of his troubles. His epilepsy was controlled.

Twelve months later, on May 2018, he presented two acute episodes of behavioral disturbance with aggressiveness and delusion that his wife and daughter were replaced by impostors (Capgras delusion), which appeared in 2 weeks. Meanwhile, a cognitive deterioration was observed with severe anterograde amnesia and psychomotor impairment. He was hospitalized in our neurology department. Cerebral MRI showed scars of former herpetic encephalitis but not new pathological changes (Figure 1B). CSF analysis showed minor lymphocytic pleiocytosis (4 leukocytes/mm^3^, normal < 3) with oligoclonal bands restricted to the CSF [more than 10 bands, type 3 according Anderson's et al. classification (7)]. Viral RT-PCRs were negative. Serum and CSF immunologic tests including those for paraneoplastic and anti-neuropil antibodies demonstrate positive anti-NMDAr in CSF only (1/10). Whole-body 18-FDG PET/CT and chest, abdomen and pelvis (CAP) CT scan were normal. A 4 h EEG didn't reveal epileptic abnormalities or focal slowing.

Levetiracetam was switched for valproid acid and the patient was first treated by risperidone, intravenous immunoglobulin (IVIG, 2 g/kg) and methylprednisolone (1 g/day for 5 days). While aggressiveness was controlled by symptomatic treatments, no improvement was seen regarding cognitive impairment, delirium and other behavioral troubles. Therefore, the patient was then treated with rituximab (1 g at day 0 and 15) and recovered slowly of his cognitive loss with a better control of his behavior troubles and was able to return home. His state remained stable after 1 year of follow-up.

### Case 2

A 57-year-old woman without significant medical history was admited in intensive care unit of our medical center in March 2018 because of a super-refractory, right, temporal, secondarily generalized, febrile, status epilepticus. MRI demonstrated a FLAIR hypersignal of mesiotemporal structure and right insula and CSF analysis demonstrated lymphocytic pleiocytosis with 234 leukocytes/mm^3^ including 95% lymphocytes, increased total proteins (1.49 g/l, normal < 0.5 g/l), decreased glucose (3.8 mmol/l, normal 3.33–4.44 mmol/l). HSV-1 herpetic encephalitis was confirmed by RT-PCR. The patient was treated with intravenous acyclovir for 3 weeks. Meanwhile, she was treated with antiepileptic bitherapy including lacosamide and valproid acid and initial sedation with midazolam and ketamin. She kept her cognitive impairment including major anxiety and behavioral stereotypies but was able to go back home on August 2018 with the help of her family. No relapse of epilepsy was noticed afterward.

Seven months later, on 09/21/2018, during rehabilityation which had led to a slow improvement but eventually good outcome, she developed paranoid troubles, delusion of incurability and that her teeth and hair disappeared (Cotard delusion), and fluctuant verbal and physical aggressiveness, which all developed in 3 days. Brain MRI only showed known lesions of the former infectious encephalitis and EEG detected few interictal epileptic abnormalities, already seen months before without any sign of worsening. Tests in the CSF performed 6 days after the symptom's onset revealed a lymphocytic pleiocytosis with 13 leukocytes/mm^3^ and a more than 10 oligoclonal bands restricted to the CSF [type 2 according Anderson's et al. classification (7)]. Anti-NMDAr IgG was positive in the CSF (1/200 in the CSF); all the other paraneoplastic and anti-neuropile antibodies were negative in serum and CSF. Viral PCR were negative in the CSF, such as syphilis and *Borrelia* serologies. Whole-body 18-FDG PET/CT, CAP CT scan, and mammography were normal. Neuropsychological evaluation showed a massive anterograde amnesia that wasn't noticed 7 months before.

Olanzapine, IVIG (2 g/kg) and methylprednisolone (1 g/day for 5 days) were started without significant improvement. After exclusion of an underlying malignancy, a second-line immunotherapy with rituximab (1 g D0 and D15) was performed. A rapid improvement was noticed, with return to his previous cognitive and behavioral state which remained constant at 1 year follow up.

The main clinical characteristics are summarized in the Table 1.

**Table 1:** Main clinical characteristics.

	**Initial herpetic meningo-encephalitis symptoms**	**Delay between herpes and AIE**	**Anti-NMDAr AIE onset**	**First line immunotherapy (IVIG + corticosteroids)**	**Second line immunotherapy (rituximab)**
Case N°1	Akinetic mutism, partial motor epilepsy	12 months	Paranoid delirium, Capgras syndrome, anterograde amnesia	Poor response	Significant improvement
Case N°2	Superrefractory right temporal status epilepticus	7 months	Paranoid delirium, Cotard syndrome, anterograde amnesia	No response	Significant improvement (back to previous state)

## Discussion

We report here two cases of long-delayed, anti-NMDAr AIE occurring several months (respectively, 12 and 7) after HSV-1 encephalitis. Those delays are the longest described so far (2–5).

We discussed the relationship between infection and AIE, questioning the probability of other causes, particularly paraneoplastic mechanism. All the investigations performed in order to find neoplasm at the time of AIE diagnosis were negative and no sign of malignancies was noticed during the follow-up, even after the immunosuppressive treatment onset. Although the direct initiation of AIE by HSV-1 cannot be formally demonstrated in our cases, especially after such a long delay, the development of this rare disease, known to be frequently post-infectious, after a cerebral infection well-known to provide anti-NMDAr AIE is intriguing. The possibility of pre-existing anti-NMDAr AIE is unlikely due to the long period of recovery and stability between infection and AIE onset.

Since 2007, post-infectious encephalitis related to anti-NMDAr antibodies have increasingly been reported, mostly after *Alphaherpesvirinae* encephalitis (8, 9), although enterovirus had been also incriminated (10). Recently, Armangue et al. described a prospective cohort of 54 HSV encephalitis followed prospectively (5). Fourteen of them (27%) developed secondary AIE—including nine related to anti-NMDAr antibodies; asymptomatic anti-NMDAr antibodies were also detected in the CSF of three other patients. Secondary AIE was diagnosed between seven and 63 days after the infection diagnosis. Thus, in this Armangue et al. cohort, all the AIE cases occurred within 2 months—a delay even shorter in patients younger than 4-years-old (median 26 vs. 43 days). The longest delay reported until now is 75 days after the infection (3).

In our two cases, initial HSV encephalitis was severe, with of our cases needing admission to our intensive care unit, and extended with super refractory partial epilepsy. In the cohort of Armangue et al., no risk factor for secondary AIE was identified among the clinical parameters of initial infectious encephalitis; only the detection of anti-NMDAr IgG in the CSF in the first 3 weeks was identified as a risk factor for secondary (although not systematical) AIE (5), an information we don't have concerning those two cases. If treatment by corticosteroids at the time of HSV encephalitis did not protect against secondary anti-NMDAr AIE even if autoantibodies were detected (5), a systematic analysis in the CSF some days after the infectious encephalitis may help identifying patients with higher risk of secondary AIE.

Interestingly, our two cases of anti-NMDAr AIE shared several characteristics. Both initially presented as encephalitis with psychiatric symptoms, including delirium with paranoid component and aggressiveness. Thus, the clinical presentation was typical for adult anti-NMDAr AIE (11) including post-infectious cases after herpes encephalitis (11). None of the symptoms can be related with the cerebral scars of former HSV encephalitis, which are not likely involved in the clinical presentation of the AIE, neither related with epilepsy reactivation. In both cases, onset was acute with psychiatric symptoms developing within 3 days. No response was observed after 3 weeks of combined first-line therapy (high-dose corticosteroids plus IVIG), whereas at least a partial improvement is usual and first-line therapy is sufficient in more than half of the cases (12). Response to rituximab as second-line therapy is usual (12).

The possibility of delayed post-infectious anti-NMDAr should be kept in mind in the long-term follow-up of patients after HSV-1 encephalitis in ensure early detection of this treatable complication. A systematic research of AIE auto-antibodies at the acute phase of infectious encephalitis may to help patient's monitoring.

## Ethics Statement

Written informed consent was obtained from the individual(s) for the publication of any potentially identifiable images or data included in this article.

## Author Contributions

GD and MB: case report concept and design, drafting the manuscript. GD, MB, CB, FB, and JP: patient's clinical and paraclinical management. GD, MB, CB, CM, FB, GM-B, and JP: revised the manuscript.

### Conflict of Interest

The authors declare that the research was conducted in the absence of any commercial or financial relationships that could be construed as a potential conflict of interest.
